# Deciphering the molecular machinery of stem cells: a look at the neoblast gene expression profile

**DOI:** 10.1186/gb-2007-8-4-r62

**Published:** 2007-04-20

**Authors:** Leonardo Rossi, Alessandra Salvetti, Francesco M Marincola, Annalisa Lena, Paolo Deri, Linda Mannini, Renata Batistoni, Ena Wang, Vittorio Gremigni

**Affiliations:** 1 Dipartimento di Morfologia Umana e Biologia Applicata, Sezione di Biologia e Genetica, Università di Pisa, Via Volta, Pisa 56126, Italy; 2 Department of Transfusion Medicine, Warren G Magnuson Clinical Center, National Institutes of Health, Central Drive, Bethesda, Maryland 20892, USA; 3 Dipartimento di Biologia, Unità di Biologia Cellulare e dello Sviluppo, Università di Pisa, Via Carducci, Pisa 56010, Italy

## Abstract

**Background:**

Mammalian stem cells are difficult to access experimentally; model systems that can regenerate offer an alternative way to characterize stem cell related genes. Planarian regeneration depends on adult pluripotent stem cells - the neoblasts. These cells can be selectively destroyed using X-rays, enabling comparison of organisms lacking stem cells with wild-type worms.

**Results:**

Using a genomic approach we produced an oligonucleotide microarray chip (the Dj600 chip), which was designed using selected planarian gene sequences. Using this chip, we compared planarians treated with high doses of X-rays (which eliminates all neoblasts) with wild-type worms, which led to identification of a set of putatively neoblast-restricted genes. Most of these genes are involved in chromatin modeling and RNA metabolism, suggesting that epigenetic modifications and post-transcriptional regulation are pivotal in neoblast regulation. Comparing planarians treated with low doses of X-rays (after which some radiotolerant neoblasts re-populate the planarian body) with specimens irradiated with high doses and unirradiated control worms, we identified a group of genes that were upregulated as a consequence of low-dose X-ray treatment. Most of these genes encode proteins that are known to regulate the balance between death and survival of the cell; our results thus suggest that genetic programs that control neoblast cytoprotection, proliferation, and migration are activated by low-dose X-rays.

**Conclusion:**

The broad differentiation potential of planarian neoblasts is unparalleled by any adult stem cells in the animal kingdom. In addition to our validation of the Dj600 chip as a valuable platform, our work contributes to elucidating the molecular mechanisms that regulate the self-renewal and differentiation of neoblasts.

## Abstract 2

Comparison of the gene-expression profiles of planarians in which all adult pluripotent stem cells (neoblasts) were eliminated and wild-type worms identified a putative neoblast-restricted gene set. This included many genes involved in chromatin modeling and RNA metabolism, suggesting that epigenetic modifications and post-transcriptional regulation are important for neoblast regulation.

## Background

Characterization of candidate genes that underpin complex biologic processes in organisms in which gene function can be studied and manipulated *in vivo *has become an important strategy. It allows fundamental issues - that are also relevant to human health and biology - to be addressed functionally. A striking example of the effective use of such approaches is in the elucidation of the molecular mechanisms orchestrating stem cell behavior *in vivo*. Much information can be obtained from *in vitro *stem cell cultures, but *in vivo *studies in mammals are problematic because they are not readily accessible for experimental analysis. For this reason, use of alternative model systems has been proposed [1,2]. Among these, we have selected freshwater planarians (Platyhelminthes), because a large number of pluripotent stem cells (so-called neoblasts) are available in adult organisms for experimental manipulation. Knowledge of the regenerative ability of these organisms is well established. The advent of molecular, cellular and genomic approaches, as well as RNA interference (RNAi) technology that can produce loss of function phenotypes, has rekindled interest in this classic model of regeneration [3,4].

Neoblasts are considered to be the only proliferating cells in asexual organism, and they can self-renew and undergo differentiation to any cell type. Thus, neoblasts are responsible for the replacement of cells lost during physiologic turnover and allow regeneration in these organisms. During regeneration, neoblasts activate a proliferation program that results in formation of a regenerative blastema, the structure from which missing parts of the body are progressively rebuilt. The distribution of neoblasts has been defined using molecular markers that allow detection of proliferating cells, such as *DjMCM2 *[5] and *DjPCNA *[6], or through BrdU incorporation [7]. These cells are scattered throughout the parenchyma with the exception of the anterior end of the cephalic region and the pharynx, and accumulate preferentially in the dorsal region, along the anteroposterior body axis.

How neoblasts maintain their pluripotency or commit to a differentiative fate remains puzzling. Recently, planarian homologs of genes such as *Pumilio *and *Piwi*, which are hallmarks of vertebrate and invertebrate stem cells [8,9], were identified, and RNAi-mediated gene silencing has indicated a function for these genes in balancing neoblast maintenance and differentiation [10-12].

Although neoblasts share a similar morphology, heterogeneity in their population has been hypothesized [3,4]. The recent characterization of *DjPiwi *(a PIWI-PAZ family member, which is specifically expressed in a neoblast subpopulation [11]) and of *Djnos *(the planarian homolog of the *nanos *gene, which is specifically found in germ line precursors [13]) supports this possibility, suggesting that subsets of neoblasts have different properties and that only some of them are true pluripotent/totipotent stem cells.

A unique advantage of studying planarian neoblasts is that these cells can be selectively destroyed by high-dose (30 Gy) X-ray irradiation [12,14-18], thus offering an opportunity to compare directly worms lacking stem cells with wild-type control organisms. This feature has been fundamental in determining the expression of newly isolated planarian genes in these stem cells. Planarian neoblasts exhibit various levels of radiotolerance, and some sub-populations appear able to survive longer after high-dose X-ray treatment [11]. Recently, we observed that planarians exposed to low-dose X-ray treatment (5 Gy) do not die, and after transection they experience regeneration delay and exhibit morphogenetic defects, and then recover. In these conditions, specific subpopulations of neoblasts survive (radiotolerant stem cells) and re-populate the planarian body (Salvetti and coworkers, unpublished data). Although several studies have focused on discovering genetic features of neoblasts during the past decade, only a few essential players have been identified, and a rigorous transcriptional profile analysis has never been undertaken.

Here we describe the production of a custom-made oligonucleotide microarray chip (Dj600 chip) that contains 600 planarian (*Dugesia japonica*) gene sequences selected on the basis of their putative involvement in process related to proliferation, migration, self-renewal, and differentiation. We used the Dj600 chip to compare the transcriptional profiles of planarians exposed to different X-ray doses (high-dose: 30 Gy [lethal]; and low-dose: 5 Gy [sublethal]) with that of untreated wild-type worms (control organisms). Our analysis resulted in the identification of a neoblast-specific transcriptional profile; we also identified genes that are involved in neoblast cytoprotection, proliferation, and migration mechanisms, which were activated as a consequence of low-dose X-ray treatment.

## Results

### Dj600 chip design

**Figure 1:**
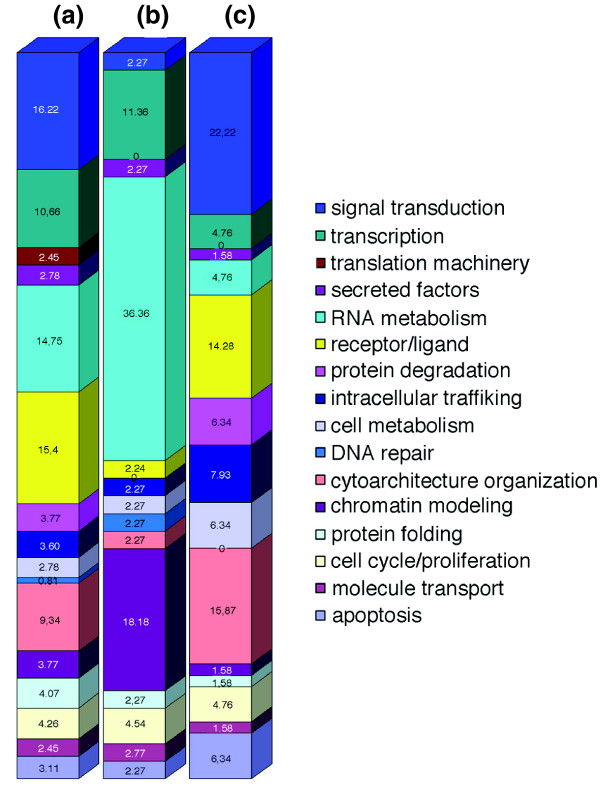
Graphical representation of the distribution (percentage) of genes by functional category. **(a) **The Dj600 chip, **(b) **genes that are downregulated as a consequence of high-dose X-ray treatment, and **(c) **gene set that is upregulated as a consequence of low-dose X-ray treatment.

The Dj600 chip (Additional data file 2) was designed using the following as sequence sources: the *Dugesia japonica *head expressed sequence tag (EST) collection produced by Mineta and coworkers [19]; previously characterized *D. japonica *sequences that are available in GenBank; and genes isolated in our laboratory. Sequences from the EST collection were searched against DDBJ/EMBL/GenBank nucleotide database using the BLASTX program. Only sequences exhibiting a significant level of homology with other known genes (e value < 10^-4^) were selected. Among those, a restricted number (612) was selected for the array printing on the basis of their putative function deduced by literature analysis. Each sequence was ascribed to a functional category (Figure 1a and Additional data file 2): apoptosis, protein folding, chromatin modeling, RNA metabolism, translation machinery, cytoarchitecture organization, cell cycle/proliferation, transcription, DNA repair, cell metabolism, intracellular trafficking, signal transduction, protein degradation, receptor/ligands, secreted factors, and molecule transport. A few sequences encoding proteins of unknown function were also included.

### Phenotype segregation by unsupervised analysis

**Figure 2:**
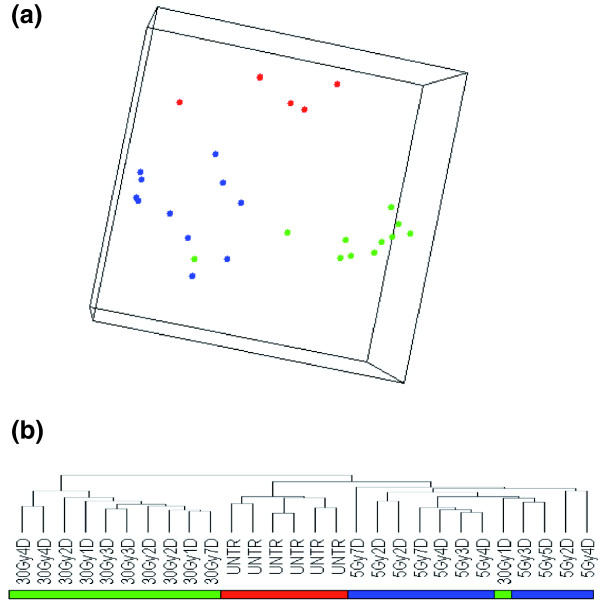
Unsupervised comparison of X-ray irradiated and control samples. **(a) **Multidimensional scaling analysis of the filtered 583 gene dataset (5 Gy samples: blue circles; 30 Gy samples: green circles; untreated samples: red circles). **(b) **Dendrogram of hierarchical clustering of all samples using centered correlation and average linkage based on the complete filtered dataset, as described in Results. The three classes of samples are indicated by the blue (5 Gy), green (30 Gy), and red (untreated) horizontal bars.

Transcriptional proximity was assessed by using the cluster program of Eisen and coworkers [20] and by multidimensional scaling (MDS). These analyses test whether the global gene expression pattern of single specimens allow them to be segregated into defined categories of particular biologic significance. Both analyses were applied to the complete dataset and were similarly successful in distinguishing between untreated control organisms, and planarians exposed to low-dose and high-dose X-ray treatment (Figure 2). The cluster analysis revealed that samples could be divided into two main groups: one for organisms exposed to high-dose X-ray treatment and one including those exposed to low-dose X-ray treatment samples and control organisms. Inside the latter group, low-dose X-ray samples cluster in a distinct subgroup that also includes a 30 Gy sample collected 1 day after treatment (Figure 2b). Although the presence in this subgroup of a 1-day sample that had undergone 30 Gy treatment may be an error in sample processing, it is important to keep in mind that 1 day is a short period after treatment. At this time, the effect of high-dose irradiation on stem cells is incomplete, and pre-existing stem cell specific mRNAs have not been completely degraded. Therefore, the gene profile in such samples may be similar to that with low-dose treatment. Although samples were clustered in the three super-groups according to treatment (untreated, and 30 Gy and 5 Gy exposures), no marked correlation was noted or molecular signatures identified that could differentiate the samples on the basis of the time of treatment.

### Supervised analysis 1: identification of stem cell specific genes

The dataset was then analyzed to test whether significant differences could be identified in gene expression between 30 Gy treated planarians (without stem cells) and control planarians (wild type). This analysis was based on 583 genes that passed filtering criteria (see Materials and methods, below) and the nominal significance level for each univariate test was set at *P *< 0.001, with a maximum false discovery rate of 10%. The class comparison identified 60 genes that were differentially expressed in 30 Gy treated samples and untreated controls.

**Figure 3:**
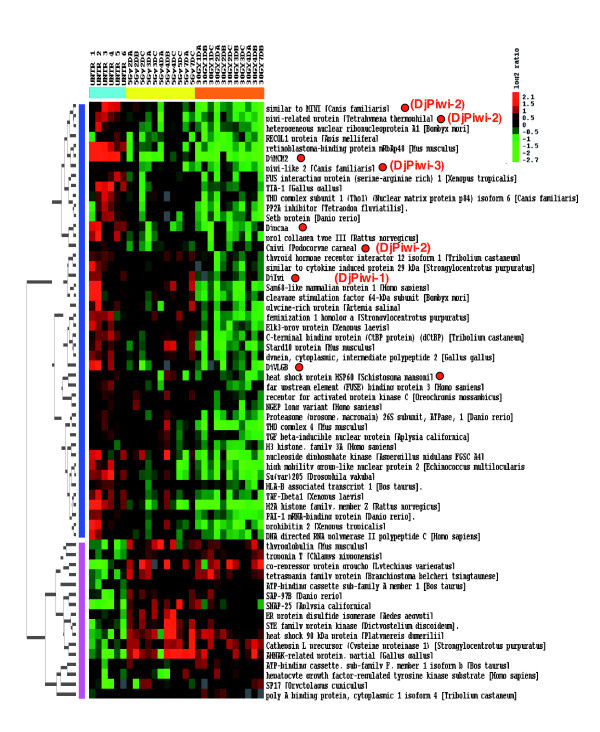
Screenshot of Eisen's clustering based on 60 genes differentially regulated in 30 Gy irradiated planarians and controls. The 60 genes are clustered unvarying the sample group (horizontal bars: cyan for controls, yellow for the 5 Gy group, and orange for the 30 Gy group). Genes are divided into two signatures: genes that were downregulated (our 'neoblast signature', blue vertical bar) and those that were upregulated (purple vertical bar) as a consequence of X-ray treatment. Genes that are known to be expressed in planarian stem cells are indicated by red dots. Clustering of experimental samples was performed according to the method of Eisen and coworkers [20]. Gene log2 ratios were average corrected across experimental samples and displayed according to the central method for display using a normalization factor, as recommended by Ross and coworkers [62]. The Tree-View software was used for the visualization. red, upregulated; green, downregulated; black, no difference.

Figure 3 shows a tree view of hierarchical clustering of significantly differentially expressed genes across all of the samples evaluated (30 Gy, 5 Gy, and controls). According to their relative expression in the sample categories, the genes could be divided in two groups; 44 genes were down-regulated as a consequence of X-ray treatment (blue group) and 16 genes were upregulated as a consequence of irradiation (purple group; Figure 3 and Additional data file 3). Samples from the 5 Gy group exhibited an intermediate and less homogeneous downregulation of genes included in the blue group as compared with the 30 Gy samples (Figure 3). Indeed, some of these genes increased in expression at 4, 5, and 7 days after low-dose treatment, whereas with high-dose treatment they were inhibited. This finding is in agreement with our data demonstrating that some neoblasts remain after low-dose X-ray treatment and initiate a rescue process, with intense proliferation about 4 days after treatment, as demonstrated by re-expression of neoblast-specific markers (Additional data file 4).

Genes that were selectively downregulated after X-ray treatment (the blue group in Figure 3) are presumably specific for planarian stem cells. Among them, genes selectively expressed in stem cells, such as *DjPiwi-1*, *DjPiwi-2 *and *DjPiwi-3 *[11], *DjMCM2 *[5], *DjPCNA *[6], *DjVLGB *[17], were identified. Regarding the distribution of these genes in terms of functional category (see above; Additional data file 2), the 44 genes principally belong to chromatin modeling, RNA metabolism, and transcription categories (Figure 1b). The distribution of these genes reveals marked changes in the relative abundance of single functional categories when compared with the sequence distribution in functional categories of the Dj600 chip (Figure 1a,b). RNA metabolism, DNA repair, and chromatin modeling are represented in the group of genes that were downregulated as a consequence of X-ray treatment by 2.5-fold, 3-fold and 5-fold more, respectively, than in the Dj600 chip. In contrast, cytoarchitecture organization, receptor/ligands, and signal transduction are reduced 4-fold, 6-fold, and 7-fold, respectively. The blue group contained no genes categorized under protein degradation and translation.

### Supervised analysis II: identification of stem cell protection mechanism related genes

Planarians subjected to low-dose irradiation (5 Gy) can re-acquire regenerative capability, because some stem cells can survive irradiation probably due to activation of some genes that are involved in stem cell protective mechanisms. To select these genes, it is necessary to eliminate the genes that are modulated in 5 Gy samples as a general response to X-ray irradiation; to this end, it is necessary to compare the transcription profiles of low-dose and high-dose X-ray treated samples. Moreover, genes that are involved in stem cell protection must be differentially regulated between low-dose X-ray treated and untreated specimens. Therefore, supervised analysis was further applied to compare 5 Gy treated planarians with 30 Gy treated and control samples. This analysis was conducted on 583 genes that passed filtering criteria (*P *< 0.001, with a maximum false discovery rate of 10%).

**Figure 4:**
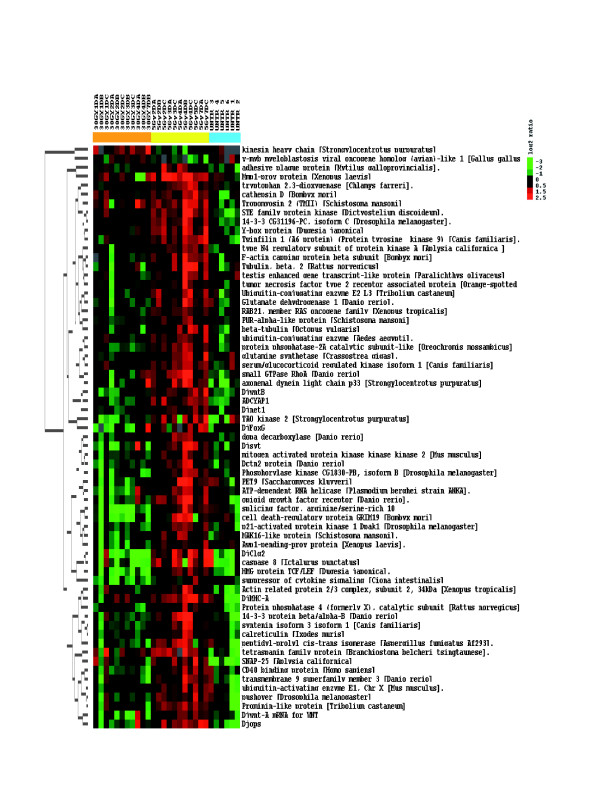
Screenshot of Eisen's clustering based on 65 genes differentially regulated in 5 Gy and 30 Gy irradiated planarians and controls. The 65 genes are clustered by sample grouping (horizontal bars: cyan for controls, yellow for 5 Gy group, and orange for 30 Gy group). Clustering of experimental samples was performed according to the method of Eisen and coworkers [20]. Gene log2 ratios were average corrected across experimental samples and displayed according to the central method for display using a normalization factor, as recommended by Ross and coworkers [62]. The Tree-View software was used for the visualization. red, upregulated; green, downregulated; black, no difference.

The class comparison identified 65 significantly regulated genes, all of which were upregulated in 5Gy treated samples but two (Additional data file 5). Figure 4 shows a tree view of hierarchical clustering of these genes across all samples. Interestingly, most of these genes reach maximal activation 4 to 5 days after X-ray treatment. The 5 Gy specific genes principally belong to the signal transduction, cytoarchitecture organization, apoptosis, intracellular trafficking, cell metabolism, and protein degradation functional categories. This distribution is rather different in terms of functional category from the distribution of genes that were downregulated as a consequence of high-dose X-ray treatment (Figure 1). Literature analysis suggests that most of the 5 Gy specific genes are functionally interconnected in a complex molecular pathway that regulates the balance between cell death and survival in response to stress stimuli or growth factors (Additional data file 6).

### Validation of the array data

To define the validity and accuracy of our global microarray analysis, we undertook two different approaches: quantitative TaqMan real-time polymerase chain reaction (PCR) for selected genes on the amplified RNA used in the array analysis; and whole mount *in situ *hybridization of selected genes in 30 Gy and 5 Gy treated planarians as well as wild-type planarians.

Ten genes that were downregulated as a consequence of X-ray treatment were selected for TaqMan real-time PCR: Gi32903884 (similar to *SLM-1 *[Sam68 like mammalian protein 1]), Gi32900731 (similar to *Rbp4 *[retinoblastoma-binding protein], Gi32901296 (similar to *H2Az *[histone family, member Z]), Gi32902158 (similar to *TAF-1-beta*), Gi32900868 (similar to *CIP-29 *[cytokine induced protein 29 kDa]), Gi13561035 (corresponding to *DjMCM2*), GiAJ865376 (corresponding to *DjPiwi-1*), Gi32899303 (similar to *Elk-*3 [ETS domain-containing protein]), Gi32904098 (similar to *REA*), and Gi 32903936 (similar to *HSP60 *[heat shock protein 60]). Also selected was one gene that was upregulated as a result of X-ray treatment, namely Gi32899321 (similar to the gene encoding AHNAK related protein). The analysis was performed on RNA obtained from 30 Gy treated and wild-type planarians (1, 2, or 7 days after irradiation).

**Figure 5:**
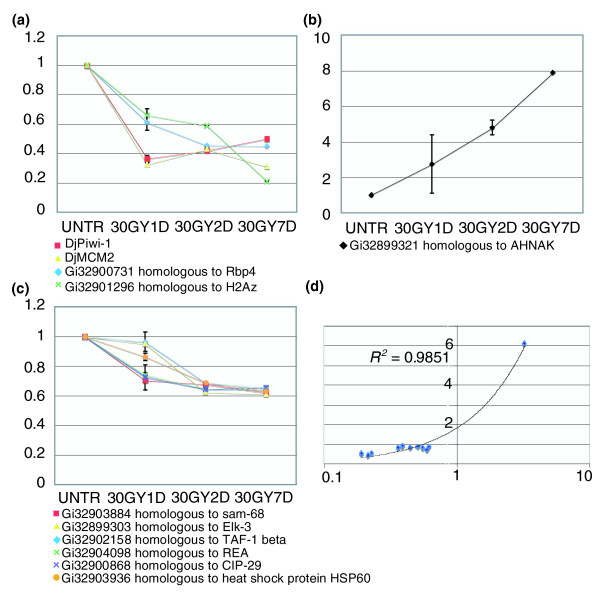
Real-time PCR analysis of expression of 10 selected genes differentially regulated in 30 Gy irradiated planarians and controls. **(a-c) **Expression levels are indicated in relative folds, assuming a value of 1 for untreated specimens (control). Values are expressed as mean ± standard deviation of six independent samples collected at each experimental condition conducted in duplicate. Genes are grouped in different charts according to the trend of their expression level in the analyzed samples. **(d) **Plot analysis of the fold-change (30 Gy versus untreated controls) measured by real time reverse transcription (RT) polymerase chain reaction (RT-PCR) versus the fold change measured on the arrays. Values are represented in logarithmic scale. *R*^2 ^= 0.9851.

**Figure 6:**
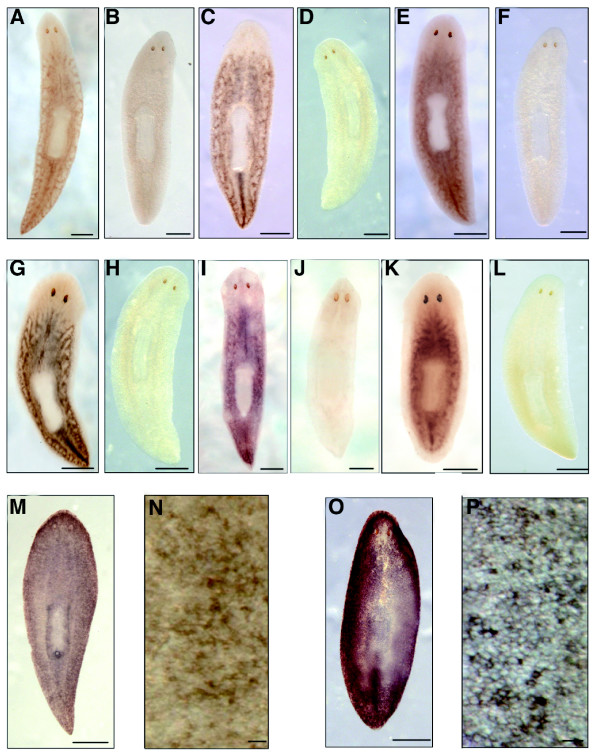
Expression of selected genes differentially regulated in 30 Gy irradiated planarians and controls (whole mount *in situ *hybridization). Expression of the expressed sequence tag (EST) clone 32900731 (*mRbAp48 *[retinoblastoma-binding protein]) in **(a) **an untreated planarian and **(b) **a planarian 6 days after 30 Gy X-ray treatment. Expression of the EST clone 32901296 (*H2A *[histone family, member Z]) in **(c) **an untreated planarian and **(d) **a planarian 6 days after 30 Gy X-ray treatment. Expression of the EST clone 32903884 (Sam68-like mammalian protein 1) in **(e) **an untreated planarian and **(f) **a planarian 6 days after 30 Gy X-ray treatment. Expression of the EST clone 32902158 (*TAF-Ibeta1*) in **(g) **an untreated planarian and **(h) **a planarian 6 days after 30 Gy X-ray treatment. Expression of the EST clone 32903936 (*HSP60 *[heat shock protein 60]) in **(i) **an untreated planarian and **(j) **a planarian 7 days after 30 Gy X-ray treatment. Expression of the EST clone 32900868 (*CIP-29*) in **(k) **an untreated planarian and **(l) **a planarian 6 days after 30 Gy X-ray treatment. Expression of the EST clone 32899321 (AHNAK-related protein) in **(m) **an untreated planarian (**(n) **magnified section showing labeled epidermal cells) and **(o) **an intact planarian 6 days after 30 Gy X-ray treatment (**(p) **magnified section showing labeled epidermal cells). Scale bars: 500 μm in panels a-m and o; 30 μm in panel n; and 25 μm in panel p.

In all cases the results obtained were consistent with the transcriptional profile detected by microarray analysis (Figure 5). The expression of some of these genes was analyzed by whole-mount *in situ *hybridization. *Sam-68*, *Rbp4*, *TAF-1-beta*, *H2Az*, *Hsp60*, and *CIP-29 *exhibited a comparable expression pattern in intact wild-type planarians, with a distribution reminiscent of the parenchymal distribution of neoblasts (signal accumulation was observed along the midline and lateral lines in parallel to a diffuse staining throughout the parenchyma posterior to photoreceptors and excluded from the pharynx). After an extended period of revelation, *TAF-1-beta *and *CIP-29 *were also detectable at low level in the central nervous system (CNS; data not shown). Expression of these genes was no more detectable 6 days after high-dose X-ray administration (Figure 6a-k). On the contrary, the *D. japonica *homolog of *AHNAK *was found at the level of the dorsal and ventral epidermis, and its expression strongly increased after X-ray irradiation (Figure 6l-o).

We also validated the data obtained in supervised analysis II by evaluating the expression of two genes, namely Gi32899629 (the planarian homolog of *HMG protein TCF/LEF*) and Gi6088097 (the planarian gene *DjSyt*), in 5 Gy and 30 Gy treated organisms, and in untreated controls. *HMG protein TCF/LEF *and *DjSyt *transcripts were upregulated 4 days after 5 Gy X-ray treatment at the level of the CNS, relative to untreated controls (Additional data file 7). As expected based on the microarray data, both *HMG protein TCF/LEF *and *DjSyt *transcripts were not upregulated after 30 Gy X-ray treatment (data not shown).

To further assess the specific neoblast expression of some of the genes found to be downregulated as a consequence of high-dose X-ray treatment we analyzed their expression in parallel with that of the known neoblast marker *DjMCM2 *(part 1) and evaluated their expression in neoblast-enriched cell fractions (part 2).

#### Assessment of specific neoblast expression of downregulated genes: part 1

**Figure 7:**
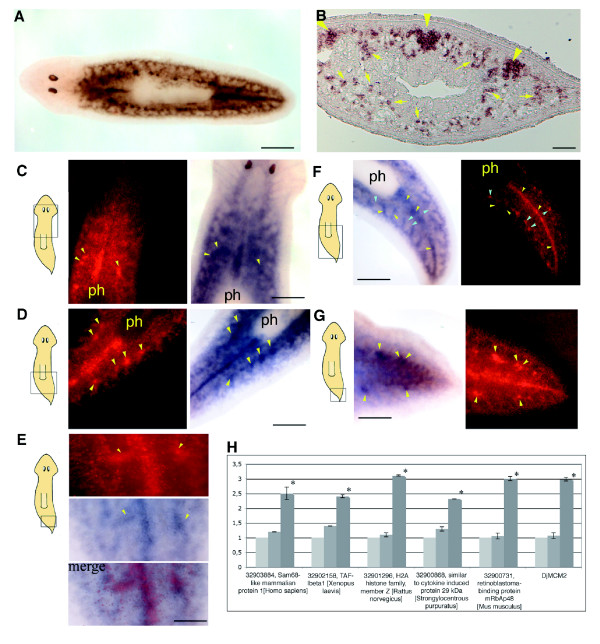
Expression of some selected genes downregulated by high-dose X-ray treatment and neoblast marker *DjMCM2 *by double *in situ *hybridization and real-time RT-PCR in neoblast-enriched cell fractions. **(a) **Expression of the neoblast marker *DjMCM2 *in an intact untreated planarian, as visualized by whole mount *in situ *hybridization. Scale bar: 500 μm. **(b) **Expression of the neoblast marker *DjMCM2 *in a transverse section of an intact untreated planarian, as visualized by section *in situ *hybridization. Arrowheads indicate the clustered neoblasts along the midline and lateral lines; arrows indicate the dispersed neoblasts. Scale bar: 50 μm. **(c-g) **Double *in situ *hybridization. Panels c-e depict the expression of *DjMCM2 *(red fluorescence) and *TAF-Ibeta1 *(NBT/BCIP blue precipitation) in the same planarian body region. Yellow arrowheads indicate some lateral patches where the co-localization of the two transcripts is particularly evident. Scale bars: 260 μm in panels c and d, and 100 μm in panel e. Panel f depicts the expression of *DjMCM2 *(red fluorescence) and *H2Az *(NBT/BCIP blue precipitation) in the same planarian body region. Cyan arrowheads indicate some lateral patches where the two transcripts do not co-localize. Yellow arrowheads indicate some regions were the co-localization is particularly evident. Scale bar: 260 μm. Panel g depicts the expression of *DjMCM2 *(red fluorescence) and *Sam68 *(NBT/BCIP blue precipitation) in the same planarian body region. Yellow arrowheads indicate some lateral patches where the two transcripts co-localize. Scarce co-localization can be observed along the midline. Scale bar: 220 μm. **(h) **Real-time polymerase chain reaction (PCR) analysis of the expression of some selected genes that were downregulated as a consequence of high-dose X-ray treatment in neoblast-enriched cell fractions. RNA was extracted from cell fractions obtained by filtration through nylon meshes of 50 μm (light gray), 20 μm (gray), and 8 μm (dark gray) pore size. Expression levels are indicated in relative folds, assuming a value of 1 for 50 μm nylon meshes. Values are expressed as mean ± standard deviation of three independent samples conducted in duplicate. The expression values of the 8 μm nylon mesh samples were compared with those of 50 μm nylon mesh samples using the unpaired *t*-test. *P *< 0.05 was considered statistically significant. *P < 0.05.

*DjMCM2 *accumulates in all neoblast subpopulations described thus far, as well as in germ-line stem cells. The *DjMCM2*-positive cells exhibit two patterns of distribution (Figure 7a,b): clustered patches of cells, accumulated on the dorsal side of the animal along midline and lateral lines; and dispersed cells, widely distributed on the dorsal and ventral parenchyma. Double *in situ *hybridizations, performed using as probes *DjMCM2 *and some selected genes that were found to be downregulated as a consequence of high-dose X-ray treatment, demonstrated that the selected genes are expressed in different subgroups of *DjMCM2*-positive cells. For example, the planarian homolog of *TAF-1-beta *exhibits a pattern of expression very similar to that of *DjMCM2 *(Figure 7c-e). The planarian homolog of the histone variant *H2Az *co-localized with *DjMCM2*, except for the cells patched along the lateral lines, in which this gene was not expressed (Figure 7f), and the majority of the analyzed organisms exhibited a faint *H2Az *signal in the clustered *DjMCM2*-positive neoblasts along the midline anterior to the pharynx (data not shown). The planarian homolog of *Sam68 *appeared to be mainly expressed in the dispersed neoblasts and only slightly detectable at the level of the clustered neoblasts (Figure 7g).

#### Assessment of specific neoblast expression of downregulated genes: part 2

Because neoblasts have a mean diameter of 7 μm, they are the smallest cells in the planarian body and are the principal cell type expected to be found after the filtering procedure through a progressive series of meshes (50 μm, 20 μm, and 8 μm). Morphologic analysis of the neoblast-enriched fraction demonstrated the presence of many small spherical cells (diameter 7 to 13 μm) with scanty cytoplasm that were specifically enriched in *DjMCM2 *transcripts [10]. Real-time reverse transcription (RT)-PCR experiments demonstrated that the expression of the selected genes that were downregulated as a consequence of high-dose X-ray treatment was significantly higher in the neoblast-enriched cell fraction than in other cellular fractions that did not pass through the 8 μm mesh (Figure 7h).

## Discussion

The unique advantage of destroying stem cells from planarians is that it offers an opportunity to compare organisms lacking stem cells with wild-type worms directly. The versatility of this model system is further amplified by the differential response of planarian stem cells to high-dose and low-dose X-ray treatment (Salvetti and coworkers, unpublished data). Here, we describe the design and application of a small-scale, high-throughput genomic tool (Dj600 chip) that is useful in retrieving information on the planarian stem cell genetic profile. Based on linear amplification of low quantities of RNA, a single planaria can provide enough material to conduct several microarray hybridizations. Hence, several individual specimens for each experimental condition represents a sample collection sufficient to obtain statistically consistent results. Hybridization of all samples, compared with a constant reference, allows us to cross-compare the gene expression profile across all of the experimental samples.

We approached the genetic profile of neoblasts by comparing planarians subjected to high-dose and low-dose irradiation with wild-type worms. According to Salvetti and coworkers [5], the high-dose of 30 Gy is lethal in *D. japonica*, whereas 5 Gy is a sublethal dose. Hierarchical clustering and MDA analysis demonstrated that the gene expression profiles identified by the Dj600 chip can discriminate between samples according to experimental treatment. As expected, high-dose X-ray treated planarians and untreated controls segregate. Although 5 Gy treated worms exhibit a profile similar to that of the control group, they represent a distinct cluster. However, none of these analyses was able to discriminate between samples according to time of collection after treatment. A possible explanation for this is that genetic changes induced by X-ray treatment occur immediately after irradiation and remain constant over 1 week (the period over which our analysis was conducted). Recently, based ultrastructural observations, we demonstrated that apoptotic cell death of neoblasts principally occurs 6 hours after high-dose X-ray treatment. Moreover, expression analysis of the stem cell markers *DjMCM2 *and *DjPiwi-1 *after 30 Gy X-ray exposure demonstrated that most of the signal was lost after 1 day [11]. On the other hand, planarians subjected to low-dose X-ray treatment also quickly lost most of their proliferating cells. Stem cells completely recover in number only about 30 days after X-ray treatment (Salvetti and coworkers, unpublished data).

Class comparison analysis between the 30 Gy group and untreated controls identified 60 differentially expressed genes. The majority of these genes (44) were selectively downregulated after treatment. Although the reduction in their expression levels could be a consequence of X-ray exposure, it is noteworthy that X-ray treatment does not affect differentiated cells, as demonstrated by using specific molecular markers [5,21]. Thus, genes that are silenced in a condition in which neoblasts are selectively destroyed are likely to be neoblast-specific genes, and these 44 genes could be considered a 'neoblast signature'. In contrast, genes that are upregulated in response to high-dose X-ray treatment, such as the planarian homolog of *AHNAK*, are not considered to be expressed in neoblasts and could encode proteins that are involved in cell reaction to stress. AHNAK is an ubiquitously expressed giant protein, which has been found to be downregulated in several radiosensitive neuroblastoma cell lines [22]. Recent data demonstrate that AHNAK is a protein that potentially influences DNA non homologous end-joining, which is the major mechanism for repairing double stand breaks in mammalian cells [23,24]. Activation of *AHNAK *expression in planarian epidermal cells might represent a cell response that culminates in activation of the DNA repair system in cells injured by X-ray exposure.

### Epigenetic modification and post-transcriptional regulation play pivotal roles in neoblast gene expression control

The genes identified in our putative neoblast signature primarily include those that are involved in chromatin modeling and RNA metabolism. Among the chromatin modeling factors, we identified the following: a putative planarian homolog of *TAF-1-beta *(SET protein); coding for a component of the INHAT (inhibitor of histone acetyl transferases) complex that strongly inhibits the histone acetyl trasferase (HAT) activity of p300/CBP by histone masking; and a homolog of a subunit of the histone chaperon NuRD compex Rbp4 (RbAp46/48). In addition to NuRD, RbAp46/48 is also a component of several other chromatin-related complexes, including Hat1, CAF-1, NURF, the Sin3 complex, and the polycomb repressive complex 2 [25]. Putative homologs coding for further chromatin modeling factors could be also found, such as the following: HP1, which, by interacting with CAF-1, is involved in defining the higher order structure of pericentric heterochromatin [26]; and CIP29, a novel, recently isolated erythropoietin (Epo)-induced protein that has a amino-terminal SAP DNA-binding motif [27]. SAP proteins regulate transcription, RNA processing, and apoptotic chromatin degradation [28,29]. Upregulation of CIP29 was found in primary human CD34^+ ^cells after incubation with thrombopoietin (Tpo), stem cell factor (SCF), and flt3 ligand (FL). CIP29 expression was also found to be greater in bone marrow than in peripheral blood, and greater in malignant cells and fetal tissues than in normal adult tissues, suggesting that it is expressed at higher levels in proliferating cells [27]. In the chromatin modeling protein group, we also identified two sequences coding for the putative planarian 'histone variants' or 'replacement histones', namely H3.3 and H2A.z. These variants might play a role in selecting specific regions or by acting as a signal that helps to recruit factors that activate or repress transcription, or both. Thus, histone variants, along with modifications to histone tails, may be involved in establishing an 'epigenetic code' [26].

Based on these data, we can hypothesize that our neoblast signature includes homologs of genes that are involved at different levels in chromatin modeling, suggesting that epigenetic modifications can be a crucial step in neoblast transcriptional regulation. Detailed analyses will be required in future studies to elucidate the specific roles played by these factors.

In addition to proteins that regulate chromatin accessibility, our neoblast signature also includes homologs of transcriptional factors that act by recruiting chromatin modeling elements. One of them is a homolog of prohibitin-2 (also known as repressor of estrogen receptor activity), which is a transcriptional repressor that acts via recruitment of histone deacetylases [30]. Another one is the homolog of the carboxyl-terminal binding protein 1, which is a member of the CtBP family - a multitask group of proteins that may function in the nucleus as co-repressors of transcription in a histone deacetylase-dependent manner and that play crucial roles in differentiation, apoptosis, oncogenesis, and development [31,32]. Interestingly, the mouse homolog of CtBP1 (mCtBP1) interacts strongly with Net (ELK3), a Ras activated transcriptional regulator whose homolog is also included in the neoblast signature. Net and mCtBP1 might recruit one of the described multiprotein complexes that contain HDAC1, HDAC2, NCoR, SMRT, Sin3, RbAp46, RbAp48, and SAP30, and other uncharacterized subunits [33-40]. Hypothetically, a similar mechanism could also exist in planarians, and a complex network including the homologs of ELK3, CtBP, and Rbp4, as part of HDAC complexes, could act together with the heterochromatin protein 1, histone variants, and other nucleosome assembly factors to define the transcriptional status of specific chromatin domains.

In our neoblast signature we also found several RNA metabolism related proteins. Among them, we identified previously characterized neoblast-specific factors such as *DjPiwi-1, DjPiwi-2 *and *DjPiwi-3*, and *DjVLGB*. Moreover, homologs of several other interesting post-transcriptional regulatory factors were also found. FUSIP1 (SRp38) cannot activate splicing, unlike other SR proteins, which constitute a family of pre-mRNA splicing factors. On the contrary, SRp38 is a potent inhibitor of splicing in extracts of mitotic cells [41]. Neoblasts are the only proliferating cells in planarians, and it is likely that expression of factors that are specifically required in mitosis is selective for this cell population. Another RNA-binding protein included in the neoblast signature is the homolog of Sam68 (Src-associated in mitosis, 68 kDa), a nuclear factor that has been postulated to play a role in cell growth control as a modulator of signal transduction and activation of RNA metabolism [42]. Among the mRNA species that bind *in vivo *to Sam68 there is the mRNA for hnRNP A2/B1 [43]. Planarian homolog transcripts for hnRNA binding protein were also found to be downregulated after 30 Gy X-ray treatment, suggesting that a network involving the homologs of Sam68 and hnRNP A2/B1 plays a role in signal transduction and activation of RNA metabolism in planarian stem cells.

Finally, our neoblast signature also includes the homolog of T-cell intracellular antigen-1 (TIA-1). TIA-1 is a RNA-binding protein that is involved in several mechanisms of RNA metabolism, including alternative hnRNA splicing and mRNA translation regulation. TIA proteins interact with FUSE-binding proteins (FBPs) - transcriptional factors that are involved in the molecular machine programming pulses of c-myc expression [44-46]. The homolog of FUBP3 (a member of the FBPs) is also included in the neoblast signature, suggesting that there is possible cross-talk between these two factors in regulating neoblast gene expression. The abundance of post-transcriptional regulation factors in stem cells is not a new discovery. In particular, neoblasts, because of their rapid response to stress stimuli (such as regeneration), retain several masked/stored mRNA molecules that allow them to initiate proliferation or differentiation programs promptly [4,47]. Ultrastructural evidence on RNA accumulation in neoblasts has also been reported. The so-called chromatoid bodies, distinctive structures of neoblasts at the ultrastructural level, are probably generated by accumulation of mRNP.

### Cytoprotection, proliferation, and cell motility pathways are activated as a consequence of low-dose X-ray treatment

Planarians exposed to low-dose X-ray treatment (5 Gy) do not die, and after transection they exhibit regeneration delay and morphogenetic defects that they recover. Preliminary results suggest that a small subpopulation of neoblasts is resistant to low-dose X-ray treatment (radiotolerant stem cells) and can re-populate the planarian body (Salvetti and coworkers, unpublished data). These radiotolerant neoblasts probably survive irradiation by activating specific genetic programs. Moreover, the entire rescue process may involve the following: release of several signaling factors from differentiated tissues; reactivation of an intense proliferation program; and active migration of stem cells to re-acquire the typical spatial organization found in untreated planarians.

To identify factors involved in these processes we compared the expression profile of the 5 Gy group with those of the 30 Gy group and the controls. Genes identified in this analysis principally belong to transduction pathway, cytoskeleton, and apoptosis functional categories. Interestingly, in an analysis of the literature we found that most of these genes have been implicated in related cell survival/death pathways that respond to different, contrasting stimuli. Some of these pathways include cell architecture reorganization and cell motility activation factors. The c-*myc *activation program leading to cell growth appears to be common to several pathways whose components were found to be upregulated in 5 Gy X-ray treated samples (Additional data file 5). Unfortunately c-*myc *is not included in the Dj600 chip because this gene has not yet isolated in *D. japonica*. Among these c-*myc *activation pathways, homolog elements of the Wnt cascade are present. The Wnt pathway has been implicated in the maintenance and self-renewal of various pluripotent stem cells and progenitor cells [48-50], and may also play a role in regenerative responses induced by chronic or acute injuries [51-53]. It is possible to speculate that low-dose X-ray treatment produces a complex inflammatory response in the planarian body. Consequently, the increased release of signaling molecules such as Wnt and activation of intracellular responsive elements will contribute to activation of survival, proliferation, and motility processes in radiotolerant neoblasts. Thus far, the only characterized planarian *Wnt *gene, namely *GtWnt5*, is expressed at the level of the CNS [54]. Although no information is available about cells that express other Wnt molecules in planarians, it is reasonable to hypothesize that the CNS may play a leading role in orchestrating the reactivation of 5 Gy resistant neoblasts.

Some of the elements found to be upregulated as a consequence of low-dose X-ray treatment appear to play contrasting roles (for instance, activation or inhibition of the same pathway). When considering this incongruence, it should be borne in mind that the rescue process that follows irradiation is a complex phenomenon including various kinds of cells, including the following: differentiated cells that activate cell cytoprotection mechanisms; neural cells that release growth factors; radiosensitive neoblasts that activate a death process; and radiotolerant neoblasts that activate proliferation or that begin to migrate and re-populate the entire body. Our planarian transcriptional profile analysis was conducted on the whole planarian body, and it is not possible to distinguish between contributions made by single cell types to the genetic profile. Further studies of the expression of single factors are necessary to ascertain their specific role.

### Data validation

That the microarray data provided here is valid is indicated by the presence in our neoblast signature of most of the previously characterized specific neoblast genes identified in this species, including *DjPiwi-1*, *DjPiwi-2 *and *DjPiwi-3 *[11], *DjMCM2 *[5], *DjPCNA *[6], and *DjVLGB *[17]. Contrary to expectation, *DjPum *- a *Pumilio *related planarian gene that is known to be expressed in planarian stem cells [10] - was not included in the list of genes identified as the neoblast signature. The most convincing explanation for this is that *DjPum *is also highly expressed in the cephalic ganglia, and this expression is not eliminated by X-ray irradiation. In addition, it cannot be excluded that CNS expression of this gene increased after irradiation. Hence, it is possible that *DjPum *is excluded from the gene group that are significantly regulated at a cutoff of *P *< 0.001.

The real-time PCR study of some genes selected from those that were downregulated following high-dose X-ray treatment confirmed that these transcripts behave as predicted by microarray analysis and are over-expressed in neoblast-enriched cell fractions. *In situ *hybridization experiments also revealed that the selected genes belonging to the neoblast signature exhibit a parenchymal expression reminiscent of that of other known neoblast markers (for instance, *DjMCM2 *and *DjPCNA*). After X-ray treatment, none of these genes was detectable under our hybridization conditions, thus confirming neoblast-specific expression. In contrast, expression of *AHNAK*, which in the microarray studies was clearly upregulated after X-ray treatment, appears markedly increased (up to eightfold) in irradiated specimens. Moreover, the expression analysis of two genes selected from those activated by low-dose X-ray treatment confirmed the results of supervised analysis II. They were upregulated 4 days after 5 Gy X-ray treatment, and their expression did not increase after 30 Gy X-ray treatment.

It is noteworthy that co-localization of the selected neoblast signature genes with *DjMCM2 *showed that these genes label subgroups of *DjMCM2-*positive cells. This is very intriguing and confirms the supposed heterogeneity of this population of cells, whose apparent pluripotency probably reflects the coordinated activities of various subtypes of stem cells.

## Conclusion

In this work, for the first time, the molecular machinery that regulates neoblast biology was evaluated by transcriptional profile analysis. To this end, we designed and validated a new, high-throughput oligonucleotide microarray platform containing 600 selected sequences, namely the Dj600 chip, which was enriched for genes that are putatively involved in stem cell related processes. The rationale underlying our sample collection was to identify stem cell restricted genes by comparing organisms lacking stem cells with wild-type worms, and to identify stem cell protective genes by comparing planarians exposed to low-dose irradiation with those exposed to high-dose irradiation and control specimens.

The Dj600 chip allowed us to retrieve some important information about planarian stem cell biology. First, we identified a group of 60 stem cell restricted genes, which we term the 'neoblast signature'. Among them, several genes appeared to be interconnected in related pathways that are involved in post-transcriptional regulation and epigenetic modification. Second, genes of the neoblast signature represent important new planarian stem cell markers that may be used in the future to identify neoblast subpopulations or to conduct functional studies to elucidate their relevance and role in neoblast maintenance/differentiation processes. Third, we also identified a set of genes that were selectively upregulated after low-dose X-ray treatment. On the basis of data presented in the literature we hypothesize that most of them act in a coordinated manner, as part of a complex network of signal cascades that are known to regulate the balance between cell death and survival in other organisms. In addition, some putative neural signaling factors were found to be upregulated as a consequence of low-dose X-ray treatment, suggesting a pivotal role for the CNS in regulating stem cell behavior. These findings pave the way to unravel the complex interconnections that occur *in vivo *between stem cells and differentiated cells.

## Materials and methods

### Animals and production of neoblast-enriched cell fractions

Planarians utilized in this work belong to the species *Dugesia japonica*, asexual strain GI [55]. Animals were kept in autoclaved stream water at 18°C and were starved for 2 weeks before being used in the experiments. Neoblast-enriched cell fractions were obtained by serial filtration as described by Salvetti and coworkers [10]. Briefly, intact planarians were dissociated into single cells by gent pipetting in a calcium magnesium free solution (2.56 mmol/l NaH_2_PO_4_·H_2_O; 10.21 mmol/l Kcl; 14.28 mmol/l NaCl; 9.42 mmol/l NaHCO_3_) containing 30 μg/ml trypsin inhibitor type II-O (Sigma, St. Louis, MO, USA). Neoblast-enriched fractions were obtained by serial filtration through nylon meshes of decreasing pore size (150, 50, 20 and 8 μm; Millipore, Bedford, MA, USA).

### X-ray irradiation

Intact planarians were exposed to 30 or 5 Gy of X-rays (200 KeV, 1 Gy/min) using a Stabilipan 250/1 instrument (Siemens, Gorla-Siama, Milan, Italy) equipped with a Radiation Monitor 9010 dosimeter (Radcal Corporation, Monrovia, CA, USA). The animals were killed 1, 2, 3, 4, 5, or 7 days after irradiation (Table 1) for RNA isolation, and 6 or 7 days after irradiation for whole mount *in situ *hybridization. Efficiency of X-ray treatment in reducing the number of stem cells was been tested by using real-time RT-PCR to determine the expression of the stem cell marker *DjMCM2*.

### Oligonucleotide probe design, printing, and postprocessing of slides

A total of 612 gene-specific 60 mer sense oligonucleotides (Tm from 79 to 88) were synthesized by Invitrogen (Carlsbad, CA, USA). Oligonucleotides were re-suspended in 3 × SSC at a concentration of 0.5 μg/μl (about 25 μmol/l) and spotted onto poly-D-lysine coated slides at the Immunogenetics Section (Department of Transfusion Medicine, Clinical Center, National Institutes of Health, Bethesda, MD, USA), with a configuration of 4 × 13 × 13 using OmniGrid robotic printer (Gene Machine, Genomic Instrument Services; San Carlos, CA). Printed slides were kept in a desiccator at room temperature for 15 days. After UV cross-linking, slides were blocked with succinic anhydride for 15 min, rinsed in distilled water, and denatured in boiling water for 2 minutes. After rapid dehydration in 95% ethanol, slides were dried by centrifugation at 800 rpm for 3 min and stored in desiccators until use.

### RNA isolation, amplification, and labeling

Total RNA was isolated from single animals (Table 1) using Nucleospin RNA II Kit (Macherey-Nagel, Düren, Germany), in accordance with the manufacturer's protocol, and RNA quality and quantity was estimated using Agilent Bioanalayzer 2000 (Agilent Technologies, Palo Alto, CA, USA) and NanoDrop (NanoDrop Technologies, Wilmington, DE, USA). Amplified antisense RNA (aRNA) was obtained from total RNA (0.1 μg) via two rounds of *in vitro *transcription, in accordance with the protocol previously described by Rossi and coworkers [11]. The fidelity of aRNA hybridization is at least equal and probably superior to total RNA for transcriptional profiling because of lack of contaminant ribosomal and transfer RNAs [56]. After amplification, the quality of aRNA was tested using the Agilent Bioanalyzer 2000. Samples from which high-quality aRNA could not be obtained were excluded. Total RNA from 50-pooled wild-type planarians was isolated by using the guanidium thiocyanate/CsCl method, and amplified into aRNA to serve as constant reference (pool of untreated organisms). the Dj600 chip contains sense oligonucleotides, 3 μg of aRNA was directly labeled by using the nonenzymatic method (ULS™ aRNA labeling kit; Kreatech, Amsterdam, Netherlands), following the manufacturer's protocol. Test and reference aRNA were labeled with Cy5 (red) and Cy3 (green), respectively, and co-hybridized onto Dj600 chip. Labeled aRNA was fragmented using the RNA fragmentation reagent (Applied Biosystems Foster City, CA, USA), in accordance with the manufacturer's instructions.

### Microarrays and statistical analyses

Hybridized arrays were scanned at 10 μm resolution on a GenePix 4000 scanner and GenePix Pro 4.0 software (Molecular Devices, Sunnyvale, CA, USA) at variable photomultipler tube (PMT) voltage to obtain maximal signal intensities with less than 1% probe saturation. Data were normalized using the 'median ratio over entire array equal to 1' method using BRB-ArrayTools software version 3.2, which is an integrated software package for array data visualization and statistical analysis developed by the Biometric Research Branch of the National Cancer Institute [57]. The spots with diameter under 25 μm, intensity under 200 in both channels, and missing values in more than 30% of experiments were excluded from further analysis. Statistical analysis of the microarray data was also performed using BRB-ArrayTools software. Unsupervised hierarchical clustering and MDS were performed to analyze the global gene expression profiles of all of the samples. Hierarchical clustering and MDS were carried out by centered correlation and average linkage. Two-sample *t*-test (with random variance model) or F test were used to identify genes that were differentially expressed between predefined classes. The criterion for a statistically significant difference in gene expression was a *P *value less than a specified threshold value (*P *< 0.001) and specified limits on false discovery, as controlled by the multivariate permutation test (90% confidence level of false discovery rate assessment; maximum allowable number of false-positive genes 10; and maximum allowable proportion of false positive genes 0.1). The number of permutations for the multivariate tests was 1,000 and for the univariant test it was 10,000. For the visualization of supervised analysis, gene log2 ratios were average corrected across experimental samples and clustering was performed using Eisen cluster program [20] and visualized using the Tree-View software (Stanford University, CA, USA).

Concordance analysis, including experimental data reproducibility and labeling bias, is routinely conducted in our laboratory when a new platform is established [58]. Reproducibility was tested using our internal reference concordance system, based on the expectation that results obtained through the hybridization of the same test and reference material in different experiments should collimate perfectly. The level of concordance was measured by periodically re-hybridizing the same arbitrarily selected test sample (30Gy4DA) with the reference sample. High concordance in gene expression predicts that ratios in different experimental conditions will be highly reproducible. With this goal, we arbitrarily divided our samples into three groups of 10 and hybridized each group on different days. Together with each group, we hybridized the 30Gy4DA sample versus the reference and the reciprocally labeled samples. At the end, we analyzed three forward and three reciprocally labeled replicate array experiments that were hybridized periodically every other 10 cDNA array slides. Standard deviations across those arrays were analyzed. This analysis demonstrated concordance level in excess of 95%. The gene expression data discussed in this report have been deposited in NCBI Gene Expression Omnibus and are accessible through GEO Series accession number GSE5318.

### Real time RT-PCR

TaqMan real-time PCR was performed using the constitutively expressed elongation factor gene *DjEF2 *as an endogenous control, as previously described [11].

Primers and probes used in the amplification reaction are listed in Additional data file 1. Relative quantification of expression of each single gene was performed using the comparative CT method, as described in the ABI Prism 7700 Sequence Detection System User Bulletin No. 2 (Applied Biosystems, Foster City, CA, USA).

### *In situ *hybridization

Whole mount *in situ *hybridization was carried out according to the protocol described by Jin and coworkers [59], with the modifications introduced by Nogi and Levin [60]. Each gene was amplified using sense and T7 promoter-adapted antisense primers, as indicated in Additional data file 1. DNA templates for *DjMCM2 *and *DjSyt *were prepared according to the method reported by Salvetti and coworkers [5]. Purified amplification products or plasmid were used to obtain DIG-labeled RNA probes using the T7 RNA polymerase (Promega, Madison, WN, USA) and the DIG-RNA labeling kit (Roche, Mannheim, Germany). Section *in situ *hybridization was performed according to the methods report by Kobayashi and coworkers [61]. Double labeling *in situ *hybridization was performed using the TSA kit (T20924; Invitrogen, Carlsbad, CA, USA), in accordance with the manufacturer's instructions. Briefly, 20 ng/ml fluorescein-labeled RNA probe produced using, as a template, various X-ray sensitive genes identified in the study were co-hybridized to the same animal with 20 ng/ml of DIG-labeled *DjMCM2 *RNA probe. Animals were then incubated with 1:100 dilution of POD conjugated anti-DIG antibodies and 1:10,000 dilution of AP conjugated anti-fluorescein antibodies. *DjMCM2*-positive cells were first detected by tyramide signal amplification, and pictures were taken under an axioplan fluorescent microscope (Zeiss). Fluorescein-labeled probes were then detected by NBT/BCIP precipitation and pictures, taken under a conventional stereo-microscope, were then compared with fluorescent images depicting the *DjMCM2 *expression pattern.

## Additional data files

The following additional data are available with the online version of this paper. Additional data file 1 lists primers and probes used in the amplification reactions for probe production and real-time RT-PCR analysis. Additional data file 2 lists the oligonucleotides included in the Dj600 chip. Additional data file 3 lists the genes identified in supervised analysis I. Additional data file 4 shows the analysis of expression of *DjMCM2*, *DjPiwi-1*, and *DjPum *transcripts by real-time RT-PCR after 5 Gy X-ray treatment. Additional data file 5 lists the genes identified in supervised analysis II. Additional data file 6 shows the involvement of several genes found to be upregulated as a consequence of low-dose X-ray treatment in cell death, survival, or motility pathways. Additional data file 7 shows the expression of two selected genes differentially regulated in 5 Gy treated planarians versus 30 Gy irradiated planarians and controls.

## Supplementary Material

Additional data file 1 Shown is a table listing the primers and probes used in the amplification reactions for probe production and real-time RT-PCR analysis. Click here for file

Additional data file 2 Shown is a table listing the oligonucleotides included in Dj600 chip. Click here for file

Additional data file 3 Shown is a table listing the genes identified with supervised analysis I. Click here for file

Additional data file 4 The image shows the analysis of the expression of *DjMCM2*, *DjPiwi-1*, and *DjPum *transcripts by real-time RT-PCR after 5 Gy X-ray treatment. RNA was extracted from untreated planarians and the animals were killed 3 or 7 days after 5 Gy X-ray treatment. Expression levels are indicated in relative folds, assuming a value of 1 for untreated specimens. Values are expressed as mean ± standard deviation of three independent samples, conducted in duplicate. Click here for file

Additional data file 5 Shown is a table listing the genes identified with supervised analysis II. Click here for file

Additional data file 6 The scheme depicts the involvement of several genes found to be upregulated as a consequence of low-dose X-ray treatment in cell death, survival, or motility pathways. Factors found in the 5 Gy specific gene set are boxed in gray; other significant factors are also indicated. The network was deduced based on a literature analysis and is a synthetic representation that does not comprise all of the involved factors. The name of the 5 Gy specific factor is indicated according to principal homology or to alternative names indicated in Additional data file 5. Click here for file

Additional data file 7 The image shows the expression of two selected genes that are differentially regulated in 5 Gy treated planarians compared with 30 Gy irradiated planarians plus controls, visualized by whole mount *in situ *hybridization in untreated intact planarians (ventral view) and in planarians 4 days after 5 Gy X-ray treatment (ventral view). Scale bar: 500 mm. Click here for file

## Figures and Tables

**Table 1:** Sample description

Code	Description	*n*	Type
UNTR	Intact untreated planarians	6	Test
30Gy1D	Intact planarians 1 day after 30 Gy X-ray exposure	3	Test
30Gy2D	Intact planarians 2 day after 30 Gy X-ray exposure	3	Test
30Gy3D	Intact planarians 3 day after 30 Gy X-ray exposure	2	Test
30Gy4D	Intact planarians 4 day after 30 Gy X-ray exposure	2	Test
30Gy7D	Intact planarians 7 day after 30 Gy X-ray exposure	2	Test
5Gy2D	Intact planarians 2 day after 5 Gy X-ray exposure	3	Test
5Gy3D	Intact planarians 3 day after 5 Gy X-ray exposure	2	Test
5Gy4D	Intact planarians 4 day after 5 Gy X-ray exposure	3	Test
5Gy5D	Intact planarians 5 day after 5 Gy X-ray exposure	1	Test
5Gy7D	Intact planarians 7 day after 5 Gy X-ray exposure	2	Test
REF	Pool of 50 intact untreated planarians	1	Reference
