# Supplementary figures and images for: Deciphering the molecular machinery of stem cells: a look at the neoblast gene expression profile
Source: Genome Biol. 2007 Apr 20;8(4):R62. doi: 10.1186/gb-2007-8-4-r62 (PMC1896013; doi:10.1186/gb-2007-8-4-r62)

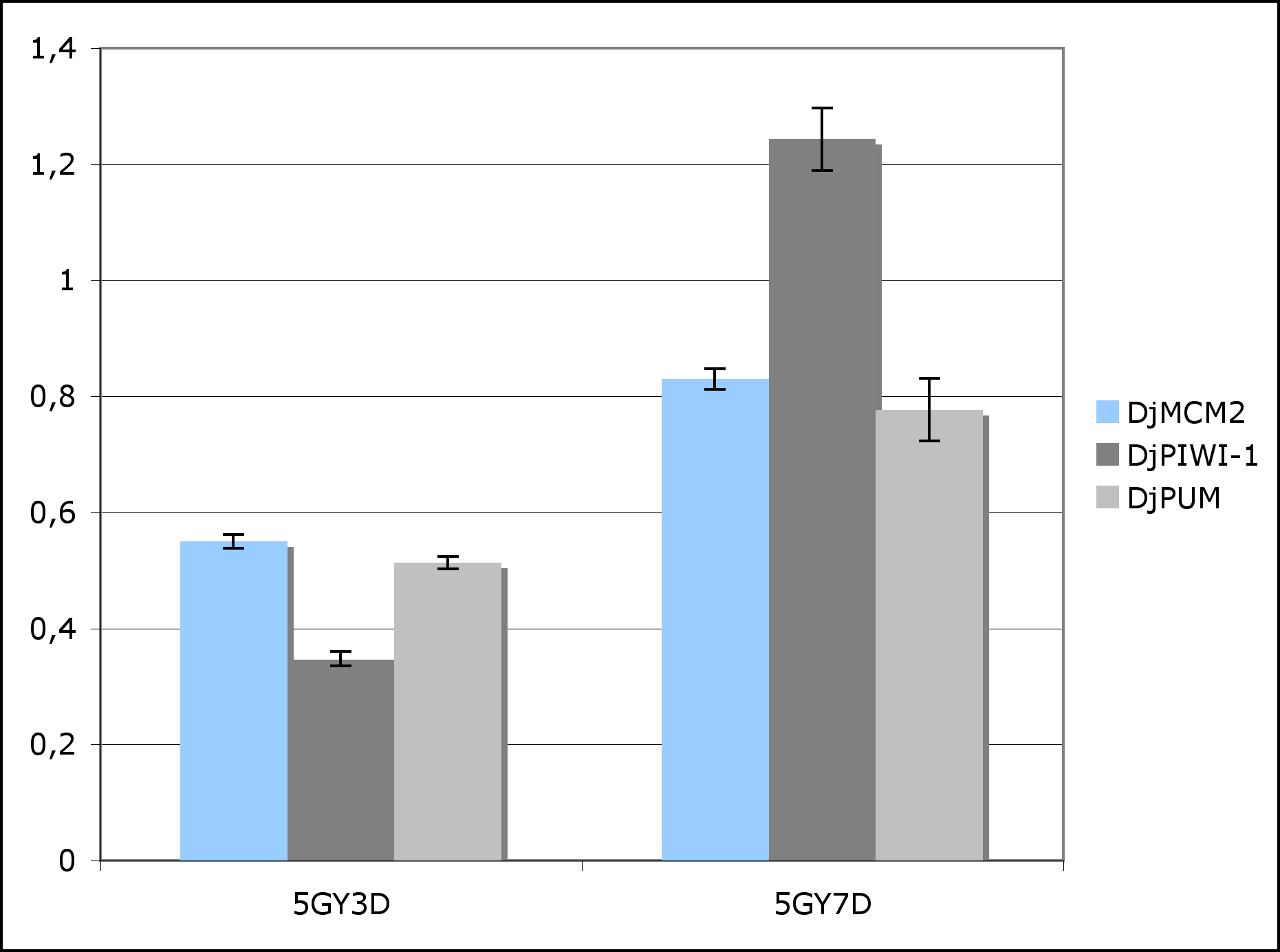

Supplement: Additional data file 4 — The image shows the analysis of the expression of DjMCM2, DjPiwi-1, and DjPum transcripts by real-time RT-PCR after 5 Gy X-ray treatment. RNA was extracted from untreated planarians and the animals were killed 3 or 7 days after 5 Gy X-ray treatment. Expression levels are indicated in relative folds, assuming a value of 1 for untreated specimens. Values are expressed as mean ± standard deviation of three independent samples, conducted in duplicate. [file gb-2007-8-4-r62-S4.tiff]

**UNTR**

**5Gy X-ray**

HMG protein TCF/LEF  
(Gi 32899629)

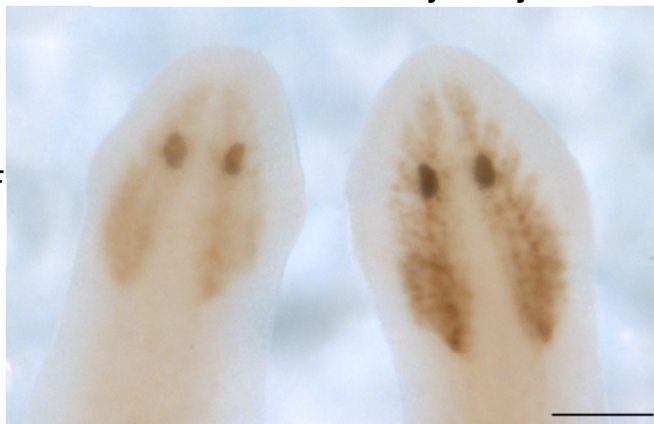

DjSyt (Gi 6088097)

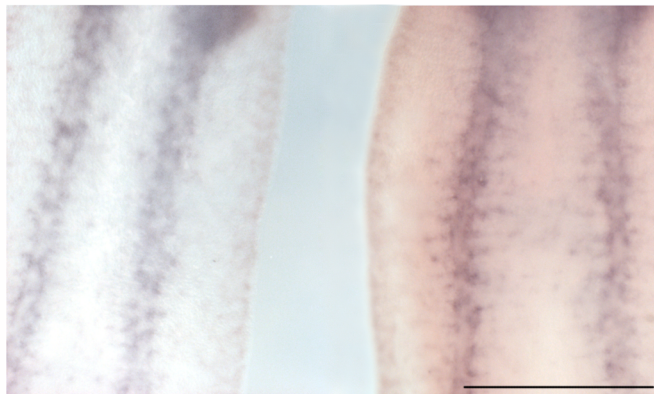

Supplement: Additional data file 7 — The image shows the expression of two selected genes that are differentially regulated in 5 Gy treated planarians compared with 30 Gy irradiated planarians plus controls, visualized by whole mount in situ hybridization in untreated intact planarians (ventral view) and in planarians 4 days after 5 Gy X-ray treatment (ventral view). Scale bar: 500 mm. [file gb-2007-8-4-r62-S7.pdf]
