# Supplementary material for: Deciphering the molecular machinery of stem cells: a look at the neoblast gene expression profile
Source: Genome Biol. 2007 Apr 20;8(4):R62. doi: 10.1186/gb-2007-8-4-r62 (PMC1896013; doi:10.1186/gb-2007-8-4-r62)
Supplement: Additional data file 6 — The scheme depicts the involvement of several genes found to be upregulated as a consequence of low-dose X-ray treatment in cell death, survival, or motility pathways. Factors found in the 5 Gy specific gene set are boxed in gray; other significant factors are also indicated. The network was deduced based on a literature analysis and is a synthetic representation that does not comprise all of the involved factors. The name of the 5 Gy specific factor is indicated according to principal homology or to alternative names indicated in Additional data file 5. [file gb-2007-8-4-r62-S6.pdf]

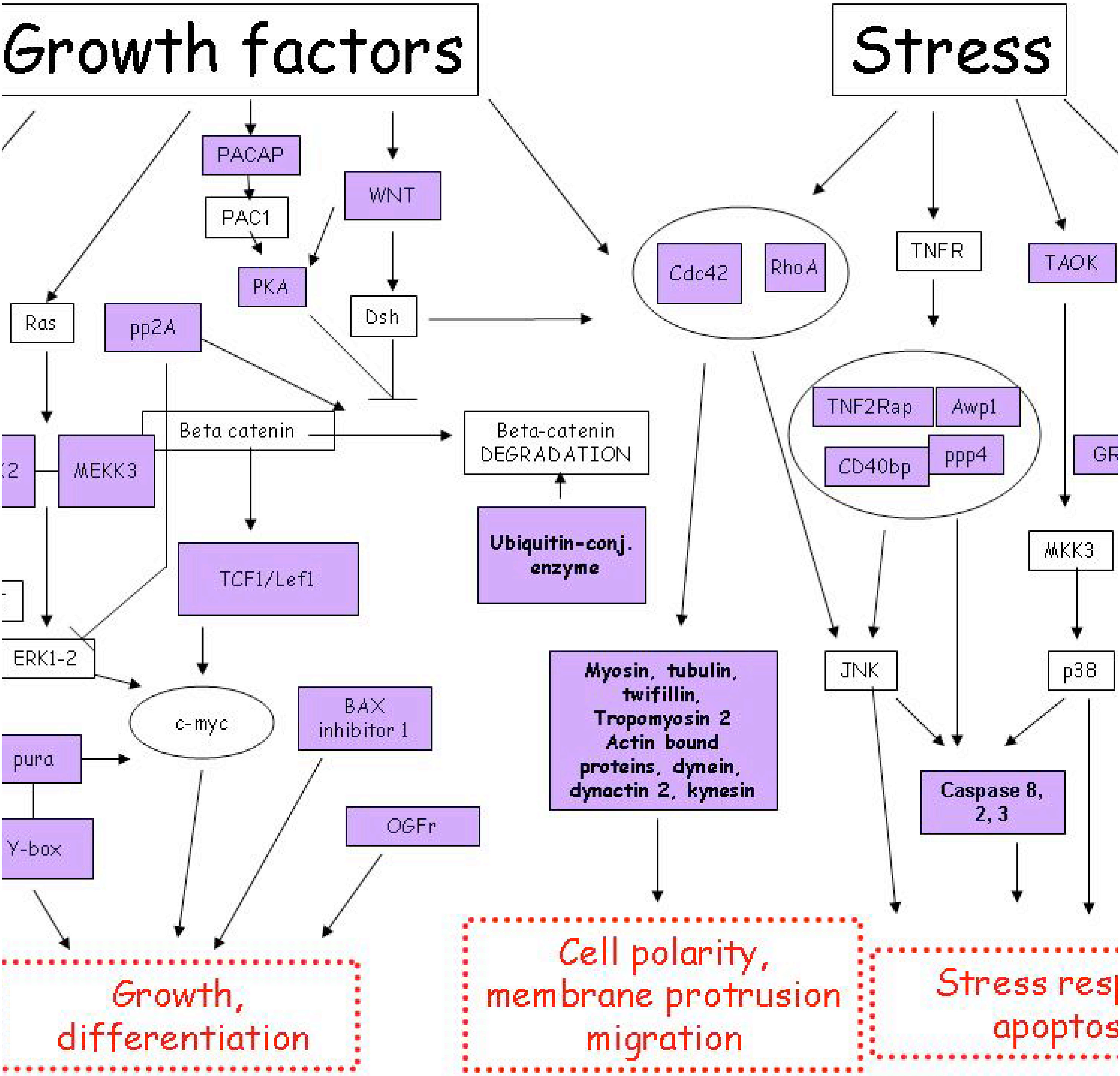
Growth factors
Stress
PACAP
PAC1
WNT
PKA
Dsh
Ras
pp2A
Beta catenin
Beta-catenin DEGRADATION
Ubiquitin-conj. enzyme
MEKK3
TCF1/Lef1
ERK1-2
c-myc
BAX inhibitor 1
pura
Y-box
OGFr
Cdc42
RhoA
TNFR
TAOK
TNF2Rap
Awp1
CD40bp
ppp4
MKK3
JNK
p38
Myosin, tubulin, twifillin, Tropomyosin 2 Actin bound proteins, dynein, dynactin 2, kynesin
Caspase 8, 2, 3
Growth, differentiation
Cell polarity, membrane protrusion migration
